# Analysis of the Drinking Behavior of Beef Cattle Using Computer Vision

**DOI:** 10.3390/ani13182984

**Published:** 2023-09-21

**Authors:** Md Nafiul Islam, Jonathan Yoder, Amin Nasiri, Robert T. Burns, Hao Gan

**Affiliations:** Department of Biosystems Engineering and Soil Science, University of Tennessee, Knoxville, TN 37996, USA; mislam50@vols.utk.edu (M.N.I.); jyoder5@vols.utk.edu (J.Y.); anasiri@utk.edu (A.N.); rburns@utk.edu (R.T.B.)

**Keywords:** animal behavior, beef cattle, drinking time, computer vision, precision livestock farming

## Abstract

**Simple Summary:**

Monitoring drinking behavior allows producers to assess the health and well-being of their beef cattle. Changes in regular drinking behavior can serve as an indicator of potential health issues. Detecting these issues early on enables the application of timely interventions, mitigating the likelihood of severe complications and enhancing the prospects for prompt and efficient treatments. In the current study, we used computer vision techniques to study and analyze the drinking behavior of beef cattle. Two different camera positions were used to identify the drinking behavior. Our proposed method was able to successfully identify both the drinking behavior and drinking time of beef cattle.

**Abstract:**

Monitoring the drinking behavior of animals can provide important information for livestock farming, including the health and well-being of the animals. Measuring drinking time is labor-demanding and, thus, it is still a challenge in most livestock production systems. Computer vision technology using a low-cost camera system can be useful in overcoming this issue. The aim of this research was to develop a computer vision system for monitoring beef cattle drinking behavior. A data acquisition system, including an RGB camera and an ultrasonic sensor, was developed to record beef cattle drinking actions. We developed an algorithm for tracking the beef cattle’s key body parts, such as head–ear–neck position, using a state-of-the-art deep learning architecture DeepLabCut. The extracted key points were analyzed using a long short-term memory (LSTM) model to classify drinking and non-drinking periods. A total of 70 videos were used to train and test the model and 8 videos were used for validation purposes. During the testing, the model achieved 97.35% accuracy. The results of this study will guide us to meet immediate needs and expand farmers’ capability in monitoring animal health and well-being by identifying drinking behavior.

## 1. Introduction

The US is the world leader in annual beef production value, and maintains the third-largest cattle herd and the largest cattle industry globally; it produced USD 86.1 billion in gross income in 2022 [1,2]. In the US, heat stress is one of the major challenges in beef cattle production and management. Due to heat stress, cattle experience health problems as well as decreases in feed intake and animal growth, which result in great economic losses to cattle farmers [3]. Based on previous studies, heat stress may also affect daily activity, including drinking and feeding [4]. Early detection of a disease is crucial because it allows for timely interventions and care based on the clinical signs and symptoms associated with that specific disease. Automatic disease detection, using advanced technologies such as artificial intelligence and machine learning, holds promise in facilitating early detection. Conventionally, direct contact (i.e., attaching sensors to animals’ bodies) has been the primary method of monitoring animal behavior [5,6,7]. However, this method faces problems such as a limited sampling frequency, low accuracy, and inconsistent readings from multiple units [8]. To help with those issues, computer vision techniques can be used to identify and classify specific animal behaviors [9,10]. Therefore, in recent years, vision-based analysis has been examined to monitor the animal behavior and health of beef cattle [8,11].

Different deep learning techniques have been developed to automate animal behavior detection methods using computer vision and video analysis technologies. Tsai et al. [12] investigated dairy cow heat stress by monitoring drinking behavior using a convolutional neural network (CNN) with an imaging system and found that drinking behavior reflects the effects of heat stress on dairy cows. Wu et al. [13] investigated a method that was proposed to detect the breathing frequency of standing resting dairy cows by using computer vision and video analysis. A Deeplab V3+ semantic segmentation model was developed using the framework of ResNet-101. Li et al. [10] studied basic motion behaviors based on cow skeletons and a hybrid convolution algorithm. The multi-resolution module was used to extract cow skeletons. The skeleton is a visual representation of a pose formed by connecting key points with lines or curves. In previous attempts, various hybrid deep learning tools were used to investigate animal behaviors. Optimizing hybrid approaches may involve fine-tuning hyperparameters to strike the ideal equilibrium between computational efficiency and model accuracy. Determining the right combination of convolution strategies can be time-consuming and resource-intensive. To simplify the tuning of hyperparameters, the pose estimation technique using different pre-trained models might be a solution. Pose estimation is a basic computer vision technique that identifies the location of a series of key body parts. In 2014, Toshev and Szegedy [14] first applied a 2D human pose estimation technique using a deep learning method. Over time, researchers developed many updated pose estimation methods such as YOLO, DeepLabCut, LEAP, and DeepPoseKit [15]. Among them, DeepLabCut is the first tool for animal pose estimation, which uses ResNet’s transfer learning to reduce the training times [16,17]. In this study, the DeepLabCut pose estimation technique was used to train and validate the model for tracking cattle body parts.

Recently, in identifying and classifying livestock activity, different artificial neural networks have been used to improve the performance of recognition tasks. Chen et al. [18] investigated pig drinking and drinker-playing behavior recognition based on ResNet50 and long short-term memory (LSTM), and the classification accuracy for the body and head regions was found to be 87% and 93%, respectively. Wu et al. [19] used a fusion of convolutional neural networks and LSTM to recognize the basic behaviors (drinking, ruminating, walking, standing, and lying) of a single cow. Nasiri et al. [20] proposed a technique to identify pose-estimation-based lameness recognition for broilers using the CNN-LSTM model. Du et al. [21] used the Resnet50-LSTM model to investigate broodstock breeding behavior, and the investigated method achieved an average accuracy of 97.32% for five types of breeding behavior recognition.

Deep learning and vision analysis play significant roles in the automated recognition of livestock behavior, as highlighted in the previously mentioned works [13]. Although researchers have identified different behaviors for various purposes, no effective method has been reported for recognizing beef cattle and/or cow drinking behavior. Identifying this behavior is crucial for detecting heat stress [22]. Consequently, as an alternative to previous behavior recognition methods, this project intended to evaluate beef cattle drinking behavior by considering skeleton-based body parts using a CNN-based model (DeepLabCut) and a time-series network (LSTM). Therefore, the specific objective of this research was to develop a computer vision system for monitoring beef cattle water drinking behavior.

## 2. Materials and Methods

### 2.1. Ethical Considerations

The University of Tennessee (UT) Animal Care and Use Committee (IACUC) approved the experiment on the use of cattle subjects under the following protocol title: Computer vision characterization of respiration as an indicator of cattle health; Protocol #: 2932-0922.

### 2.2. Experimental Site

The experiment was conducted at the University of Tennessee, Middle Tennessee AgResearch and Education Center (MTREC), between 5 October 2022 and 12 October 2022. The experiment involved four purebred black Angus cattle, all of which were 1 year old at the time. The cattle were kept in a barn equipped with a roof and automatic fans that activated when the temperature reached predefined conditions, ensuring they were protected from direct sunlight, rain, wind, and muddy conditions when needed. An open space was connected to the barn, offering plenty of room for the animals to move about without restraint. Surrounding both the open space and the barn were wooden fences, ensuring that the animals remained within their designated boundaries. MTREC staff diligently supervised the well-being and health of all animals during the experiment, adhering to the farm’s standard operating procedures and following veterinary recommendations.

**Figure 1:**
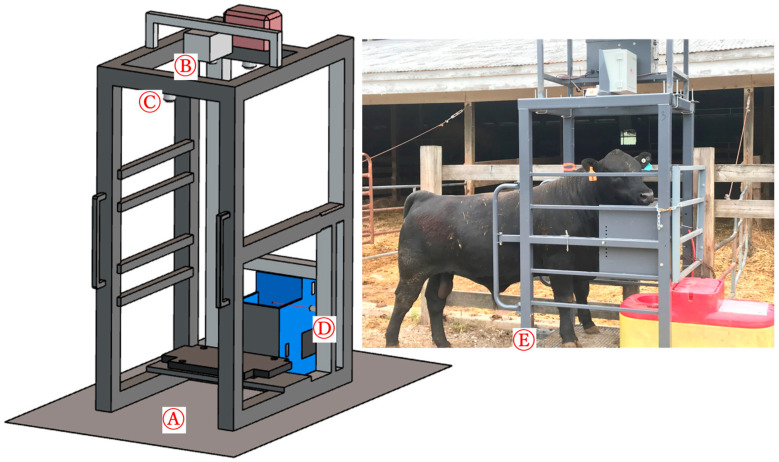
Structure of the vision system: (**A**) mainframe, (**B**) camera unit, (**C**) first ultrasonic sensor (trigger the camera), (**D**) second ultrasonic sensor (for ground truth), and (**E**) animal entering the waterer.

The waterer was placed in the open space to provide free access to drinking water. The vision system was installed over the top of the waterer. The vision system consisted of a mainframe, a ball waterer, and a sensing camera unit (Figure 1). The sensing camera unit included a Raspberry Pi 4 microprocessor, a Raspberry Pi camera (8MP IMX219, Arducam Technology Co. Limited, Kowloon, Hong Kong, China), and two ultrasonic sensors. System power was provided using Power over Ethernet (PoE). To optimize power usage, an ultrasonic sensor (Figure 1C) was used to trigger camera recording when an animal was in the field of view of the vision system. The camera was positioned at a height of 1.8 m above the ground. Another ultrasonic sensor was used to monitor the drinking behavior of the animal. This ultrasonic sensor was mounted on the side of the waterer at a height that was just above the ball of the waterer. The operating principle was that when the animal was pushing the ball to drink water, the ultrasonic sensor would detect the animal’s head, thus confirming drinking behavior.

### 2.3. Data Collection and Annotation

**Figure 2:**
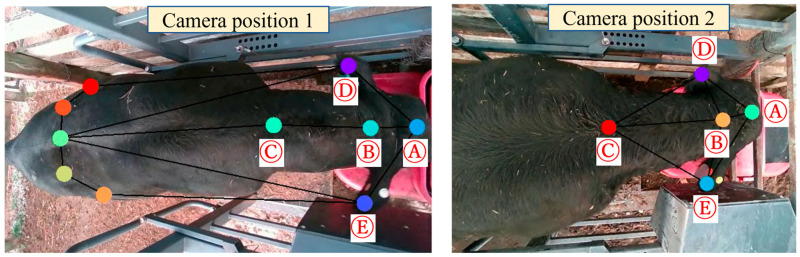
The annotated key points and beef cattle pose skeleton: (**A**) head, (**B**) upper neck, (**C**) lower neck, (**D**) left ear, and (**E**) right ear.

In this study, animal drinking behavior was recorded from two camera positions. This was to ensure that our algorithm was not sensitive to a specific camera position and could potentially handle flexible camera mounting options. A total of 78 videos, including 39 videos of each position, were recorded. The videos were recorded as H264 files. After that, the MP4 (RGB) and Hierarchical Data Format (HDF) files were extracted from each H264 file. The MP4 (RBG) files were used to track the key body points. A total of five key points were used to observe the pose skeleton (Figure 2). Each skeleton line that connects two key body points represents the relationship between the two points. The skeleton lines were defined in the training dataset to help with model accuracy. An open-source ‘VGG Image Annotator’ online annotator tool was used to label the drinking time for the recorded video as ground-truth data [23]. The second ultrasonic sensor (Figure 1D) was also used to collect the ground-truth drinking time for each animal. The two types of ground-truth data were compared to ensure the accuracy of the labeled data.

### 2.4. CNN-Based Pose Recognition

**Figure 3:**
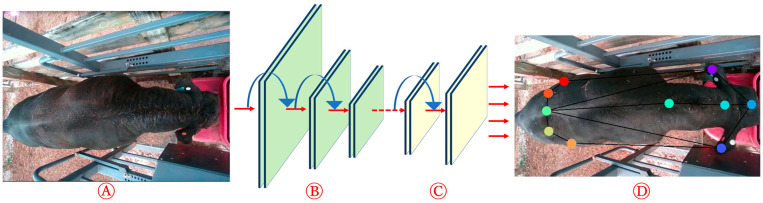
The diagram outlines the pose estimation workflow: (**A**) input, (**B**) pre-trained model (ResNet50), (**C**) deconvolutional layers, and (**D**) output with pose skeleton.

Based on the literature review, key body points and pose were identified using the DeepLabCut CNN-based pose estimation tool [16]. DeepLabCut has two ResNet (50 and 101) architectures to choose from, and ResNets facilitate the substitution of deconvolutional layers with dense layers to enhance the process of feature extraction [24]. The network has the capability to learn labeled key body points, which allows greater probabilities of recognition and reduced likelihoods of misidentifying other points. In the last stage, the trained model analyzed the videos and estimated the pose for the whole dataset. In this study, the pre-trained ResNet50 architecture was utilized as the transfer learning approach for pose estimation. Figure 3 demonstrates the diagram that outlines the pose estimation workflow.

### 2.5. LSTM-Based Drinking Behavior Estimation

**Figure 4:**
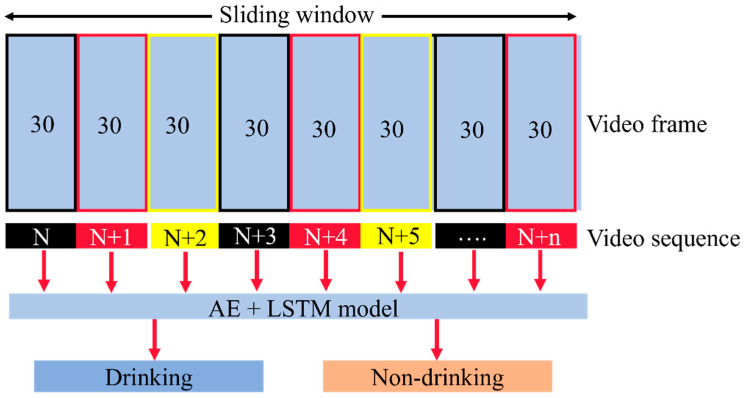
The schematic diagram of the sliding-window sampling technique.

The LSTM-based model was used to classify drinking and non-drinking behavior. This model used the output from the previous DeepLabCut model, which were the coordinates of the key body parts, as the input, and produced either drinking or non-drinking classes as the output. The input included coordinates from 30 consecutive frames (1 s of video). In this study, 30-frame sequences were constantly sampled and predicted, and a 30-frame step size was set to make sure that the video sequence was repetitive each time. Therefore, after setting the fixed window size to 30 and sliding the window with a step size of 30, the proposed LSTM algorithm was used to detect the drinking behavior from small video segments obtained from the sequence. Figure 4 shows the schematic diagram of the sliding-window video sequence sampling technique in this study.

**Figure 5:**
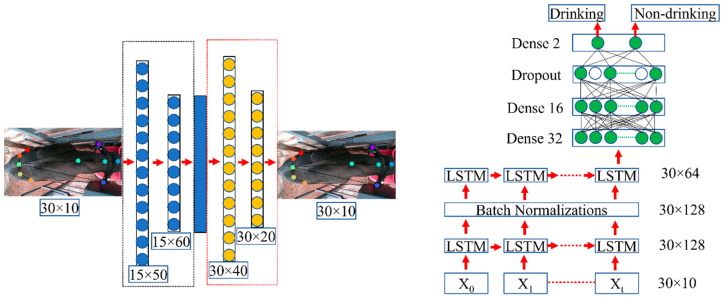
The architecture of the proposed AE (**left**) and LSTM models (**right**).

In deep learning studies, the augmentation of data is a very useful method to enhance the efficiency of the model training process by reducing over-sampling and augmenting random transformations [20]. Therefore, before starting the LSTM training, a convolutional autoencoder (AE) was applied to augment the training dataset. Figure 5 shows the architecture of the proposed AE and LSTM models. For the AE, the entire training dataset was randomly split into two sets (train and test) at a proportion of 8:2. The selected AE model was trained for 300 epochs. The mean absolute error loss function and Adam optimizer were used for the AE model. After applying the augmentation process, the proposed LSTM model was trained for 1000 epochs, including a cross-entropy loss function and Adam optimizer. The learning rate and learning rate decay were used to train the LSTM model, set at 1 × 10^−5^ and 1 × 10^−7^, respectively. The input dimension of the LSTM was 30 × 10, which represented 30 frames × 5 key points in each frame × 2 coordinates (x and y) per point.

Evaluation indices including accuracy, precision, recall, specificity, F1 score, and AUC were used to validate the proposed algorithm. The evaluation indices were calculated as shown in Equations (1)–(5) [20]:

(1) $$Accuracy=\frac{TP+TN}{FP+FN+TP+TN}$$

(2) $$Precision=\frac{TP}{TP+FP}$$

(3) $$Recall=\frac{TP}{TP+FN}$$

(4) $$Specificity=\frac{TN}{TN+FP}$$

(5) $$F1\;score=2\times \frac{Precision\times Recall}{Precision+Recall}$$

where TP = true positive, TN = true negative, FP = false positive, and FN = false negative.

## 3. Results and Discussion

### 3.1. Pose Estimation

**Figure 6:**
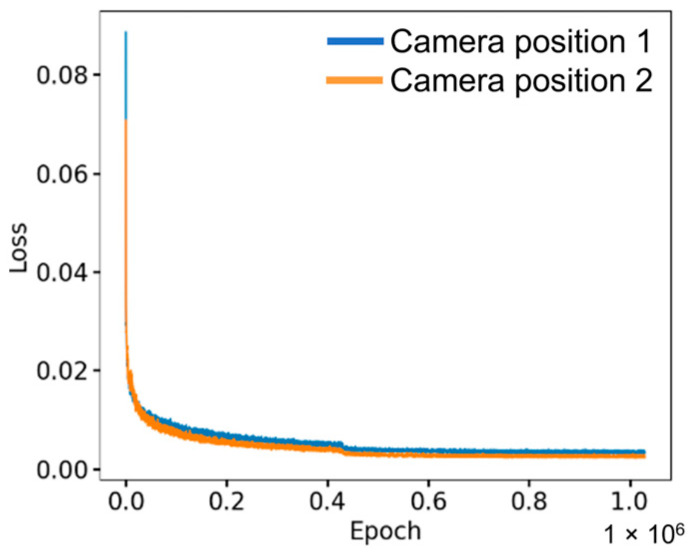
The loss values throughout the DeepLabCut model training for two camera positions.

Figure 6 shows the loss values of the DeepLabCut-based model for identifying key points. The training procedure took nearly five days for each model, and the selected weights of the DeepLabCut were attained at 1,030,000 iterations along with loss values of 0.0033 and 0.0023 at the learning rate of 0.001 for camera positions 1 and 2, respectively.

### 3.2. Evaluating the AE and LSTM Model Performance

**Figure 7:**
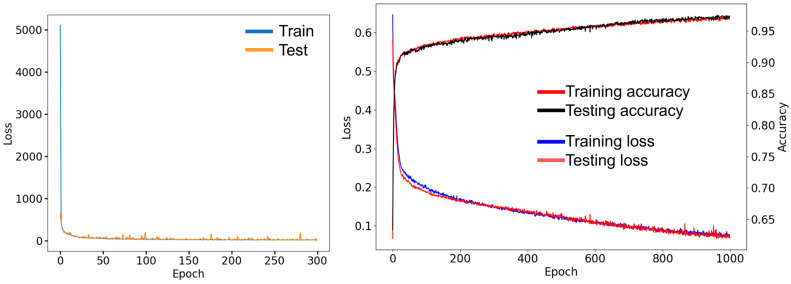
The changes in loss values during the AE model (**left**), and loss and accuracy values during the LSTM model (**right**).

Figure 7 (left) indicates the AE model loss values. The loss values for the best model were 17.90 and 11.77 for training and testing, respectively. There was a decreasing trend in the loss values with increasing epoch numbers. After epoch 150, the training and testing loss curves were stabilized, which indicates that the proposed AE model has gained sufficient convergence. Figure 7 (right) shows the performance of the proposed LSTM model. After 200 epochs, the accuracy increased, and the loss decreased simultaneously. The accuracy values for the best LSTM model were achieved at 97.35% and 97.37% for training and testing, respectively.

Eight 1-minute-long individual videos were used for validation of the proposed LSTM model. The videos were collected from both camera positions. Table 1 shows the validation results of the proposed LSTM algorithm. Our evaluation includes a range of metrics, including efficiency, precision, recall, specificity, F1 score, AUC, and confusion matrices (as shown in Supplementary Materials, Figures S1–S8), to comprehensively assess the model’s performance. The highest accuracy was obtained at 98% for video numbers 1, 4, 5, 6, and 7. The lowest accuracy was obtained at 95% for video number 2. In addition, this proposed algorithm was able to calculate the drinking and non-drinking times.

### 3.3. Comparison of Different Related Studies

Efforts to develop automated dairy cattle drinking behavior models have been underway by various research groups. Most researchers were mainly focused on automatic basic behavior recognition methods including for drinking, ruminating, walking, standing, and lying. Among them, Tsai et al. [12] developed an imaging system for monitoring and analyzing drinking behavior. When a dairy cow head was detected in the drinking area, the drinking status and the duration of drinking were recorded until the dairy cow left the drinking area. A YOLOv3 model was used to detect the head movements and a recognition accuracy of 90% was achieved. Wu et al.’s [19] CNN-LSTM network was proposed to recognize the basic behaviors of a single cow. The developed algorithm mainly included two parts. The VGG16 framework was first used as the network skeleton to extract the feature sequence corresponding to each video. The second part was basic behavior recognition using the designed LSTM model. The combination of the two parts formed the final output to realize the recognition of basic behaviors. Shu et al. [25] developed a video system for monitoring different behaviors (drinking, eating, lying, and standing). YOLOv5 architectures were trained using the transfer learning method. The results showed that the recognition accuracy of drinking behavior was 97.50%. Zhang et al. [26] proposed a SlowFast-based cow behavior recognition algorithm to identify cow behaviors such as standing, lying down, walking, drinking, and eating. The SlowFast algorithm was designed to address the challenges of recognizing actions and events in videos. The key idea behind the SlowFast architecture was to tackle the temporal resolution trade-off problem in video action recognition. In videos, actions can occur at different speeds, and capturing both fast and slow temporal dynamics is essential for accurate recognition. The accuracy of drinking estimation was found to be 92.60%.

Table 2 summarizes the outcomes of the computer vision models for cattle drinking behavior recognition from previous research. Currently, there has been limited research on cattle drinking behavior recognition. Most studies to date have applied computer vision models to recognize some basic behaviors by training the image for a particular action. Detecting drinking needs recognition of different actions including the motion of the head and the initial movement of drinking. Some of the previous research did not focus on specifically drinking actions. Due to those limitations, the previous methods of drinking recognition accuracy were lower than for the other behaviors. In the current study, we used a combination of a video annotator (head movement) and an ultrasonic sensor (drinking status) to produce the ground-truth model.

In contrast to the previous research, the proposed method in this study was a rapid and non-invasive technique. The DeepLabCut pose-estimation-based model accurately categorized the drinking behavior and could analyze drinking time tracked through videos with different lengths. Addressing the continuous detection and tracking of key points on constantly moving cattle, without the need for expert pre-marking, is an essential challenge. In this study, the focus was on tracking the key points of interest in the head area. DeepLabCut has the capability to effectively acquire body part information, even when faced with challenges such as a complex and changing background, uneven lighting conditions, or distortions caused by the camera. DeepLabCut also offers several key benefits, including cost reduction in manual behavior analysis, achieving high accuracy with a minimal number of training images, and eliminating the necessity of placing visible markers on specific locations of interest [16]. Creating a DeepLabCut model entails a potentially intricate and resource-intensive procedure. It necessitates a robust GPU, and the training phase may consume a substantial amount of time, particularly when dealing with large datasets. This can create challenges for individuals who have restricted computational capabilities.

## 4. Conclusions

This study proposed a skeleton-based computer vision method for beef cattle drinking behavior recognition. It used cameras from different positions and orientations for beef cattle pose estimation using DeepLabCut with a ResNet50 backbone. A dataset containing 70 videos was evaluated using the model to create the sequential key body points data. After that, an LSTM model was used to classify the drinking and non-drinking behaviors. The accuracy of the model was 98.25%, which was higher than the previous studies using other computer vision methods. We are currently conducting a preliminary study focused on addressing a significant and practical challenge within the field of livestock farming. This research has the potential to be applied to various livestock species, including but not limited to beef cattle, sheep, horses, and more. By employing the same vision system and pose estimation techniques, we can additionally quantify the respiration rates of animals. Currently, our group is collecting more video data from different farms using different camera positions. More collected data will further increase the accuracy of the LSTM model as well as different classification models. They will also help prepare the vision system as a practical tool for beef farms worldwide.

## Figures and Tables

**Table 1:** Validation results of the proposed LSTM algorithm.

Video Number	Accuracy (%)	Precision (%)	Recall (%)	Specificity (%)	F1 Score (%)	AUC (%)	Drinking Time (s)	Non-Drinking Time (s)
1	98.25	100.00	95.24	100.00	97.56	97.62	20	37
2	96.49	97.50	97.50	94.12	97.50	95.81	40	17
3	94.74	100.00	90.32	100.00	94.92	95.16	28	29
4	98.25	100.00	97.62	100.00	98.80	98.81	42	15
5	98.28	100.00	97.30	100.00	98.63	98.65	36	22
6	98.28	100.00	94.44	100.00	97.14	97.22	17	41
7	98.28	100.00	92.86	100.00	96.30	96.43	13	45
8	96.56	95.12	100.00	89.48	97.50	94.74	41	17

Video numbers 1 to 4 were recorded at camera position 1, and 5 to 8 were recorded at camera position 2.

**Table 2:** Comparison of computer vision model outcomes for cattle drinking behavior from previous research and the current study.

Dataset	Model	Recognition Accuracy (%)	Reference
Dairy cow image	YOLOv3	90.00	[12]
Dairy cow video	VGG16 and LSTM	95.00	[19]
Dairy cow video	YOLOv5	97.50	[25]
Dairy cow video	Slowfast	92.60	[26]
Beef cattle video	DeepLabCut and LSTM	98.25	Current study

## Data Availability

Data are presented in this article in the form of figures and tables.

## Supplementary Materials

The following supporting information can be downloaded at: https://www.mdpi.com/article/10.3390/ani13182984/s1, Figure S1: Confusion matrix for video number 2; Figure S2: Confusion matrix for video number 2; Figure S3: Confusion matrix for video number 3; Figure S4: Confusion matrix for video number 4; Figure S5: Confusion matrix for video number 5; Figure S6: Confusion matrix for video number 6; Figure S7: Confusion matrix for video number 7; Figure S8: Confusion matrix for video number 8.
