# Supplementary material for: Analysis of the Drinking Behavior of Beef Cattle Using Computer Vision
Source: Animals (Basel). 2023 Sep 21;13(18):2984. doi: 10.3390/ani13182984 (PMC10526023; doi:10.3390/ani13182984)
Supplement: Supplementary file 1 [file animals-13-02984-s001.zip › animals-2603683-supplementary.pdf]

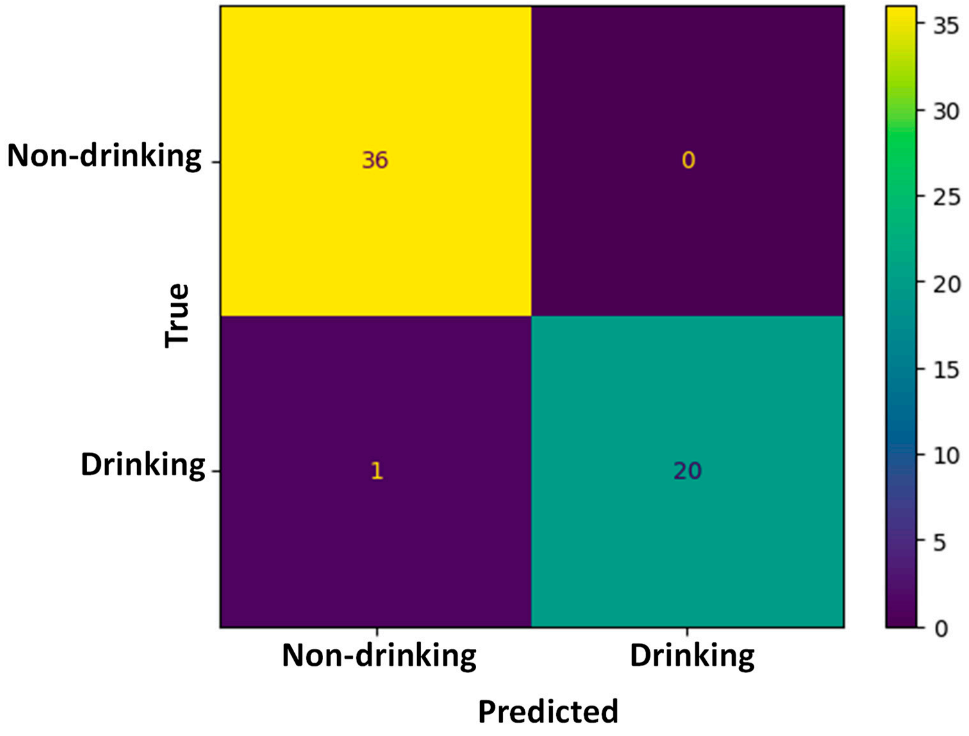

Figure S1. Confusion matrix for video number 1.

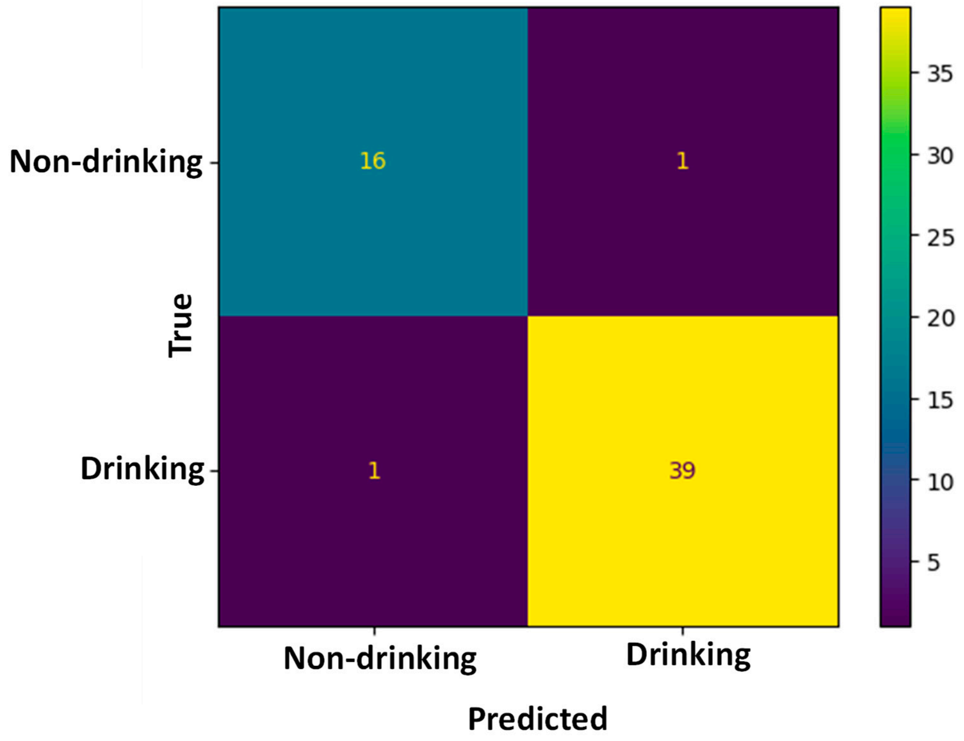

Figure S2. Confusion matrix for video number 2.

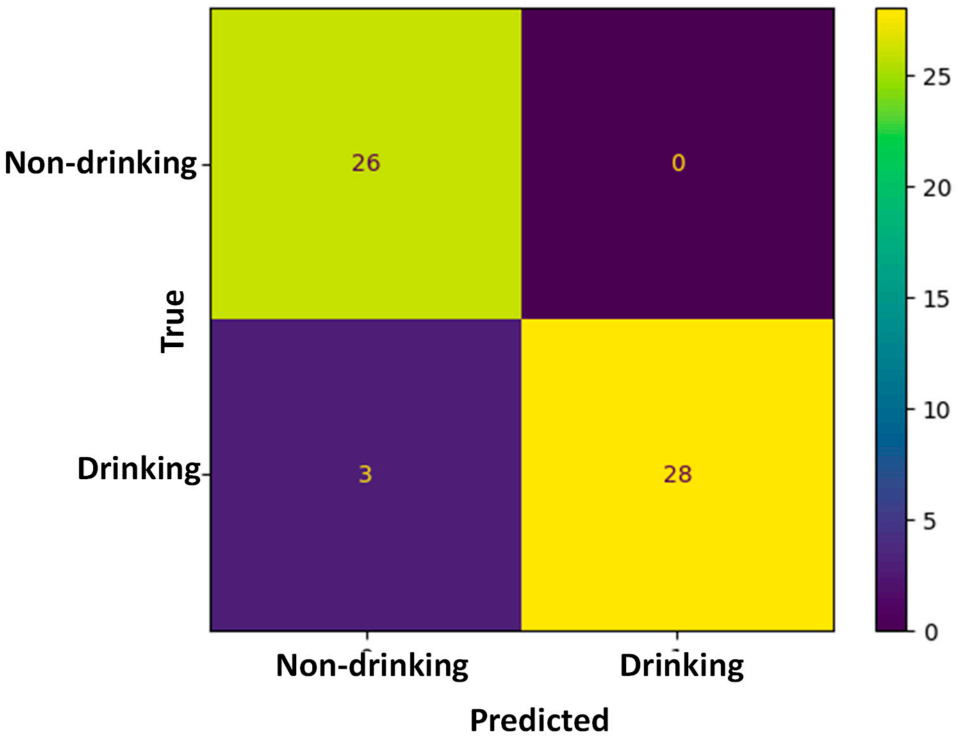

Figure S3. Confusion matrix for video number 3.

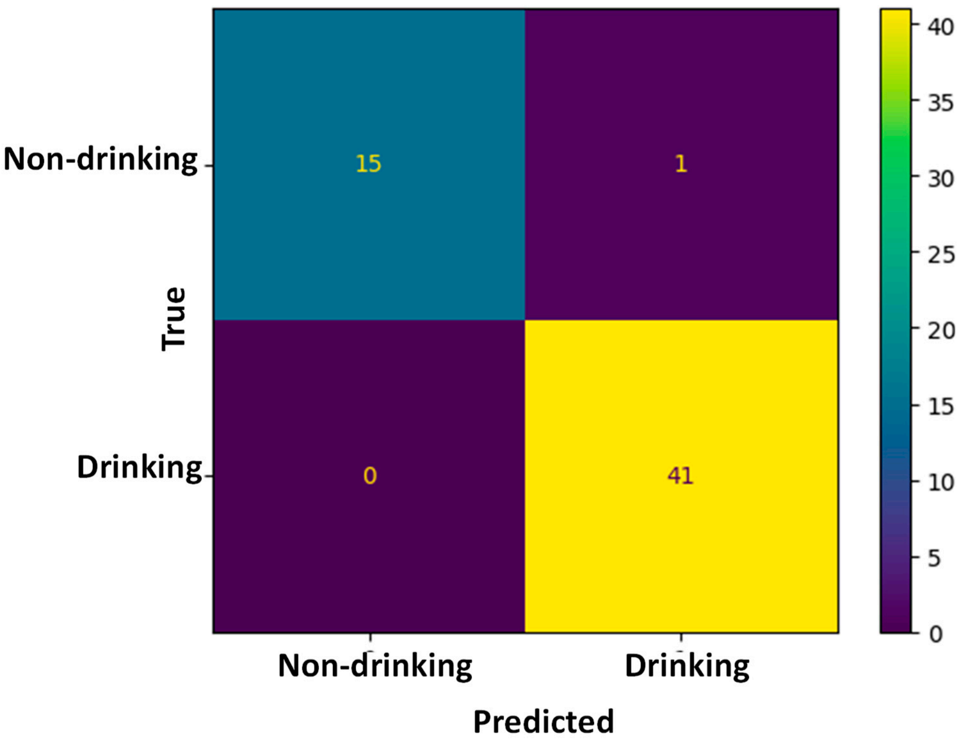

Figure S4. Confusion matrix for video number 4.

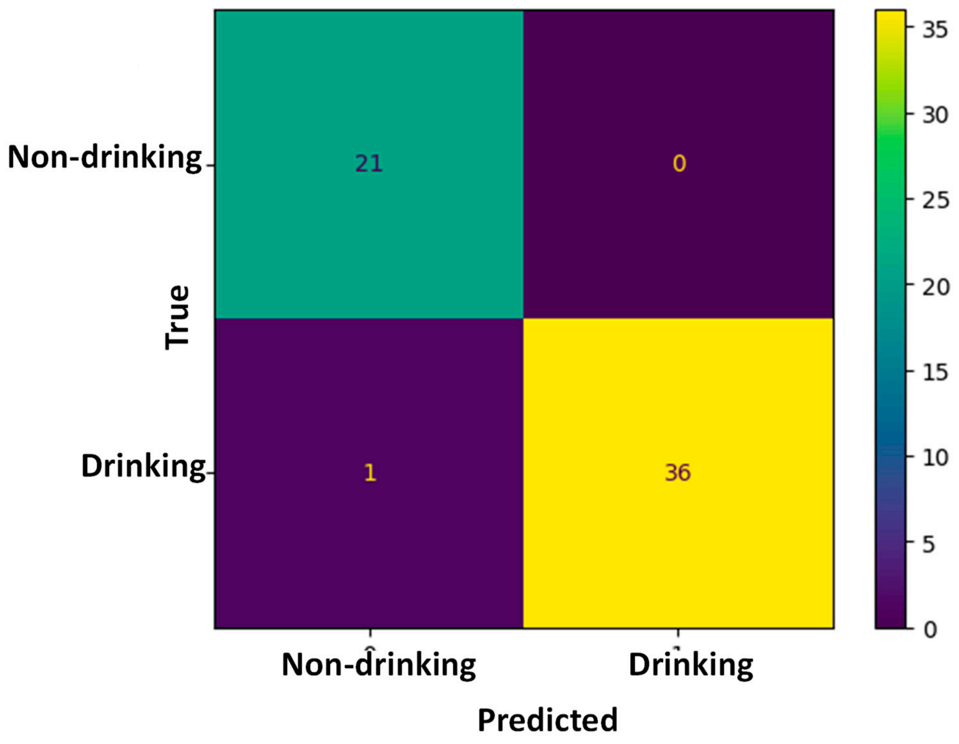

Figure S5. Confusion matrix for video number 5.

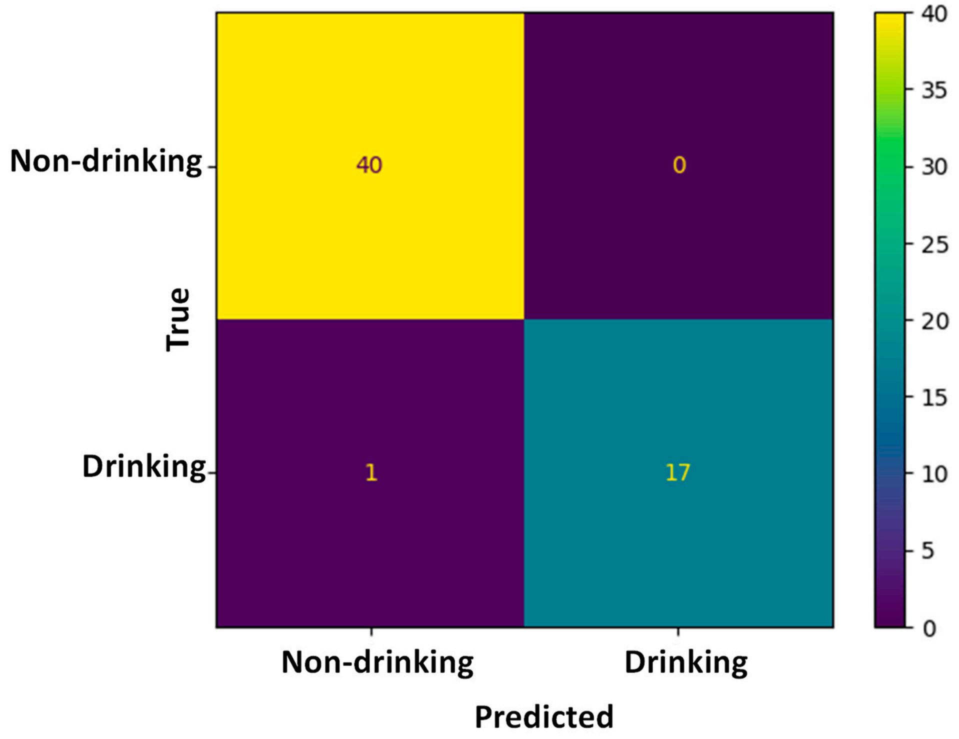

Figure S6. Confusion matrix for video number 6.

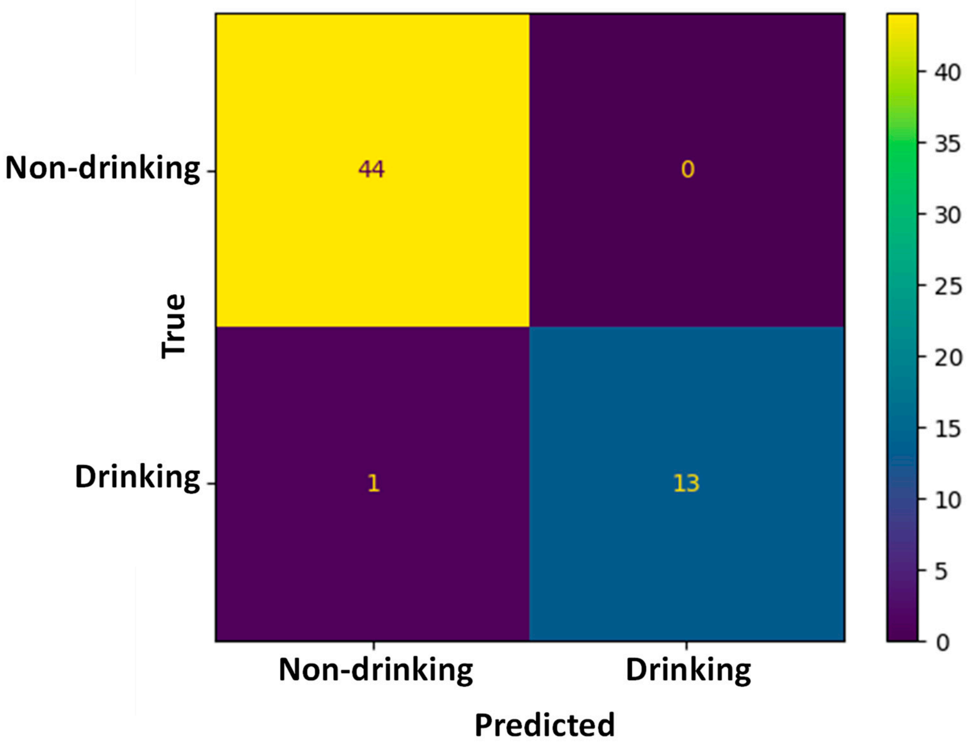

Figure S7. Confusion matrix for video number 7.

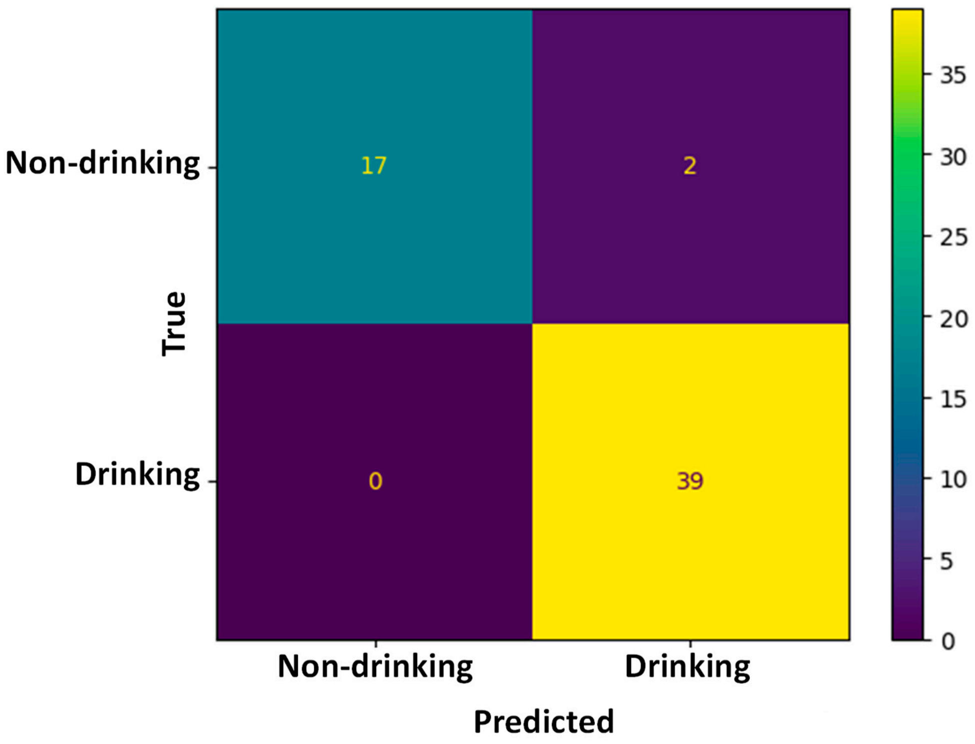

Figure S8. Confusion matrix for video number 8.
